# Evolution of Foamy Viruses: The Most Ancient of All Retroviruses ^†^

**DOI:** 10.3390/v5102349

**Published:** 2013-09-25

**Authors:** Axel Rethwilm, Jochen Bodem

**Affiliations:** Universität Würzburg, Institut für Virologie und Immunbiologie, Versbacher Str.7, Würzburg 97078, Germany; E-Mail: jochen.bodem@vim.uni-wuerzburg.de

**Keywords:** foamy viruses, retroviruses, hepadnaviruses, evolution, genetic conservation, recombination

## Abstract

Recent evidence indicates that foamy viruses (FVs) are the oldest retroviruses (RVs) that we know and coevolved with their hosts for several hundred million years. This coevolution may have contributed to the non-pathogenicity of FVs, an important factor in development of foamy viral vectors in gene therapy. However, various questions on the molecular evolution of FVs remain still unanswered. The analysis of the spectrum of animal species infected by exogenous FVs or harboring endogenous FV elements in their genome is pivotal. Furthermore, animal studies might reveal important issues, such as the identification of the FV *in vivo* target cells, which than require a detailed characterization, to resolve the molecular basis of the accuracy with which FVs copy their genome. The issues of the extent of FV viremia and of the nature of the virion genome (RNA *vs*. DNA) also need to be experimentally addressed.

## 1. Introduction

Retroviruses have gained a lot of general interest, because of the devastating human AIDS pandemic over the last 35 years, making HIV one of the best-studied viruses of all times. However, lentiviruses, such as HIV, are not the most widespread of *Retroviridae*. The most prevalent of all retroviruses (RVs) are probably spumaretroviruses (or foamy viruses), a subfamily of RVs that has attracted altogether a group of approximately only 250 researchers from 10–15 labs worldwide over the same period of 35 years. 

This life in a scientific niche is also illustrated by the fact that while the PubMed database lists more than 330,000 items for the search criteria “HIV or AIDS”, the hits are only around 1,000 for “foamy virus”. The interest in foamy viruses (FVs) is increasing, since these viruses offer unique opportunities for RV research, due to their distinct replication strategy, distinguishing them from orthoretroviruses [1,2,3], in clinical applications as potential retroviral vectors [4,5,6], due to their non-pathogenicity in any species [3,4], and as a model virus for studying antiretroviral drugs for HIV-1, since FV integrase has been crystallized [7,8,9]. However, the particular molecular characteristics of a virus may be a reflection of its evolutionary success or failure. Additionally, in terms of evolutionary success and genome stability, no RV compares to FVs, as suggested by a number of recent studies.

## 2. FV Sequence Conservation

Viruses with an RNA genome or an RNA (pre-) genome phase in their replication cycle are likely to accumulate genetic changes over time [10,11]. Individuals infected chronically by such viruses generally present (if untreated for their infection) a dynamic swarm of genetically slightly different viral variants at any given moment [10,11,12]. In virology, this has been termed the quasispecies concept [10,11,12]. At the basis of this concept is the error-prone replication by the RNA polymerase or reverse transcriptase (RT) together with a high *in vivo* replication rate [10,11,12]. The errors due to these types of mutations can be in the range of 1:10^3^ to 1:10^5^ point mutations per replication cycle [10,11]. 

This scenario may be further complicated by recombination events exchanging larger pieces of genetic information mainly between related viruses by replicase during genome copying [10,11]. Recombination events by template switching are well known for several virus families, such as picornaviruses, and have been investigated extensively (for a review, see [13]). Furthermore, in the case of viruses with segmented genomes, such as orthomyxoviruses, the exchange of whole segments may alter the features of the progeny substantially [10,11,12,13]. It is evident that genetic recombination or gene exchange (reassortment) *in vivo* requires the infection of one cell within one host by at least two viruses. 

Numerous examples of high genetic variability of the retroviral, hepadnaviral and positive (+) strand RNA virus families have been investigated in detail (HIV, hepatitis B virus (HBV) and hepatitis C virus (HCV) are only the most prominent ones causing chronic infections in humans [14,15,16,17,18,19,20,21,22,23,24]), while only a handful of examples for recombination were described for the non-segmented minus (−) strand RNA viruses [25].

However, FVs appear to be an exception to these general rules of RNA virus and retrovirus genetic variability, because their genome is highly stable [26,27,28,29,30]. The *in vivo* variation of FV genomes has been estimated to be around 1.7 × 10^−8^ substitutions per site per year, which was quite close to that of mitochondrial DNA of 1.16 × 10^−8^; these values are unprecedented among viruses having an RNA phase in their replication cycle [28]. 

**Figure 1:**
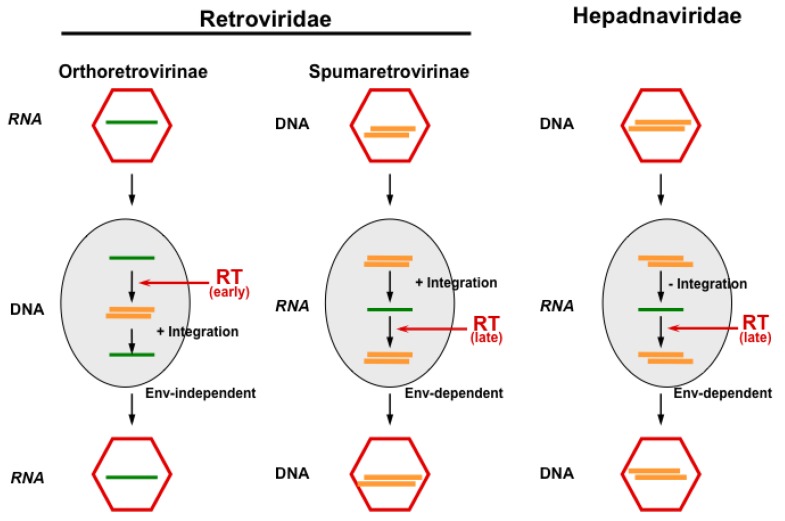
The principal replication strategies of viruses making use of reverse transcription. While orthoretroviruses are RNA viruses, which replicate through a DNA intermediate and require integration for reproduction, hepadnaviruses are DNA viruses replicating through an RNA intermediate without the integration of their genome. FVs appear to functionally bridge these pathways, since they reverse transcribe (at least to a significant extent) late in replication (like hepadnaviruses) and must integrate their genome into the host cell genome (like orthoretroviruses). Furthermore, cellular exit of FV particles depends on their cognate glycoprotein as in hepadnaviruses, while budding of orthoretroviral particles is Env-independent (Figure adapted from [3]). RT, reverse transcriptase.

Even hepadnaviruses, which are related to FVs in terms of their replication pathway (Figure 1), show a one thousand-fold higher *in vivo* point mutation rate upon chronic human infections [16], despite the fact that they have a much more compact genetic order with largely overlapping reading frames that puts constraints on the variability of hepadnaviruses [31]. Indeed, the common origin of retroviruses with hepadnaviruses has been suggested long ago [32,33], and FVs might represent this evolutionary link, from which both viral families evolved [34,35,36]. However, fossil viral records are not available to address this question. Thus, for the time being, the relation of FV genomes to orthoretroviruses and hepadnaviruses is more or less a functional one. Therefore, due to the accuracy of genome copying and sequence stability, an FV sequence can be used to determine the animal subspecies of its origin, provided the exclusion of *trans*-species infection [37]. This feature has practical consequences, for instance, by designing breeding strategies for non-human primates (NHPs). For obvious reasons, it is often impossible to obtain blood samples from wild or quasi-wild animals, particularly the great apes. However, methods have been adapted from experiences in AIDS-research to amplify FV sequences from fecal samples obtained without disturbing the animals [38]. Due to the enormous genome conservation of FVs, such analyses revealed a phylogenetic co-distribution of the viruses with their hosts [37].

Investigations of whether the exceptionally high processivity, observed for the FV RT enzyme [39], reflected by the accuracy of *in vitro* genome copying and, thus, possibly an intrinsic feature of the FV RT, revealed a surprising observation: analysis of bacterially-expressed protein indicated that the fidelity of the FV RT enzyme approximated the one of HIV RT in a similar assay [40]. The overall mutation rates were 1.7 × 10^−4^ for prototype FV (PFV) and 7.5 × 10^−5^ for HIV, respectively [40]. Two thirds of PFV RT errors were due to deletions or small insertions [40]. Apparently, many more deletions or insertions were discovered for the FV enzyme than for the HIV RT. These *in vitro* findings resembled, in some way, deletions observed in the long terminal repeats (LTRs) upon FV amplification in cell culture [41,42].

**Figure 2:**
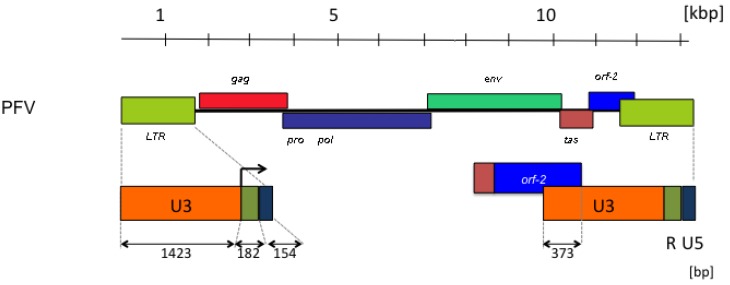
Prototype foamy virus (PFV) genome organization with particular reference to the length and structure of the long terminal repeat (LTR) with the canonical U3, R and U5 regions. The retroviral genes (*gag*, *pro-pol*, *env*) are abbreviated as usual. The FV accessory *tas* and *orf-2* reading frames are shown, as well as the spliced reading frame, giving rise to the Bet protein. The start of transcription in the LTR is indicated by an arrow in the enlargement at the bottom. The numbers refer to lengths in kilobase pairs (kbp) or base pairs (bp).

The length of the LTRs is a striking feature of FV genomes (Figure 2). The U3 regions contribute to these by approximately 85%. In particular, the LTRs of FVs from primates have extraordinarily long U3 regions of more than 1,400 bps [41]. Approximately one third of the U3 regions of primate FVs are protein-encoding *bet* sequences (Figure 2). The possible functions of the sequence remainder, aside from relatively short DNA motifs required for the regulation of gene expression [43,44], are inadequately defined and need further characterization. Because the rest of the genome has protein-encoding and *cis*-acting RNA or DNA functions, or both [2,45,46], the deletions in the U3 LTR region may be tolerated by virus replication in cell culture [41,42]. Such deletions were actually found upon obtaining proviral FV molecular clones from higher primates [47], and the detection of the undeleted LTRs [41,48] delayed their initial identification [49,50]. 

Another deletion that also occurs *in vivo* [41,48] is located in the coding region of the *tas* gene and contains the DNA copy of an almost full-length viral RNA, except approximately 200 bps, that includes the splice, which is normally needed to encode the Bet protein [51,52]. Since Tas is an essential viral accessory protein that exerts its function in *trans* [49,53], the so-called ∆Tas genome behaves like a defective-interfering particle [2,3].

The low fidelity detected by the *in vitro* study of the FV RT prompted the analysis of viral replication in cell culture [54], similar to investigations correcting the initially-reported very low *in vitro* fidelity of HIV RT [55,56]. The determination of the FV RT error rate on a lacZ-gene fragment that also was used *in vitro* [40,54] revealed that the frequency of deletions now dropped to insignificant values, and that of miss-incorporations, due to point mutations, was now found to be around 4 × 10^−4^ per site and replication cycle [54]. The vast majority of the point mutations were G –> A transitions, suggesting a role of APOBEC3 proteins in shaping the FV genome sequences (see below) [57]. 

The FV accessory Bet protein has been identified to counteract cellular APOBEC3 proteins [58,59]. However, the prototype FV (PFV) isolate Bet is inefficient compared, for instance, to the Bet protein of feline FV (FFV) proviral plasmids in this respect ([60], M. Löchelt, personal communication). Probably this inefficiency, partially due to the adaptation of the PFV isolate to cell culture conditions, is responsible for some misleading findings on Bet function [61]. Furthermore, it remained unclear whether this inefficiency to counteract APOBEC3 or the rather low cellular *bet* gene expression levels obtained with the particular expression plasmid [54,62] or both reasons led only to an around 50% reduction of G –> A mutations identified in the target cells transduced by FV vectors [54]. In contrast, normally, the *bet* gene is highly expressed in naturally FV-infected cells [63,64,65].

It should be noted that even if one ignores all G –> A mutations, by assuming that Bet will prevent these in the *in vivo* situation, such an “idealized” FV RT error rate of around 1.1 × 10^−5^ point mutations per replication cycle would still remain [54]. This is significantly more than observed for FV *in vivo* replication and indicates that more constraints act on the virus that do not allow for *in vivo* genome variability. This difference may be due to the potential presence of a factor that negatively influences the accuracy of FV genome copying in the HEK 293T cells, used to produce the vector particles, or the cells lack a factor that positively regulates the FV RT fidelity, which is present *in vivo*. However, the amount of virus produced by transfection of HEK 293T cells (usually on the order of 10^6^ virions/mL of supernatant) is probably much higher than *in vivo* values, although these await a more detailed characterization [66,67]. 

The “idealized” FV mutation rate is even higher than that of primate T-cell leukemia viruses (PTLVs), which influences infected cells to multiply and, thus, amplify the viral genome by proviral expansion, thus avoiding an RNA phase [68,69]. With respect to sequence conservation, it is worth mentioning that PTLVs also show weak evidence of human adaptation and human viruses in a given area resembling their simian virus counterparts in the same area more than human isolates from a different geographic region [70]. It has been suggested that PTLVs are not in need of a genetic adaptation to the human host, since they appear to be readily adapted [70]. The multiple *trans*-species transmissions of simian PTLVs to humans represent a comparable situation to FVs [70]. However, PTLVs are transmitted among humans, while FVs are apparently not [3,70]. 

Compared to the persistent infections observed with HIV, HBV and HCV, FVs give rise to a rather low *in vivo* replication rate in blood and most tissues [48,66,71]. This low *in vivo* replication might be in part responsible for the observed low FV mutation rate; however, it should be noted that the mutation rate in cell culture was calculated per replication cycle and the *in vivo* evolutionary mutation rate per year. However, the low FV viremia and the different scales of mutation rate measurements probably do not represent the whole story, because in the SFV system, active viral replication has been found in cells of the oral mucosa [66,67], which is consistent with the relative ease of isolating virus from NHPs by throat swaps [72]. Thus, the constraints put on the virus *in vivo* cannot currently be reproduced by cell culture experiments, and much more work is needed to understand the FV replication in greater detail. In particular, the identification of the exact target cells should be followed by a detailed characterization of the process of foamy viral reverse transcription. 

## 3. FV Recombination

As noted above, one requisite for virus recombination to occur in the offspring is the infection of one host cell by at least two distinct, but genetically-related, viruses; although, a so-called illegitimate recombination event between genetically unrelated sequences can occur, but seems to be very rare compared to a homologous recombination [10,11]. However, if it takes place, it can have really bizarre and unpredictable results. In RV replication, recombination is an obligatory event: during reverse transcription, the RT enzyme has to switch RNA templates in order to synthesize the double-stranded cDNA genome. From HIV-infection, circulating recombinant forms (CRFs) are known that significantly contribute to virus resistance and diversity [23,73]. It has been estimated that HIV RT crosses its template at a minimum of 2.8-times per replication cycle [74]. 

Given the distinct replication pathways and probably different *in vivo* target cells between orthoretroviruses and spumaretroviruses, a recombination event between these viruses belonging to distinct retroviral subfamilies appears very unlikely. Furthermore, such a nota bene illegitimate non-homologous recombination event has not been reported from the supposedly thousands of NHPs dually-infected with lentiviruses and FVs in NHP centers. 

However, in the unlikely event such a new virus develops, such a new kind of “FV” may acquire the pathogenic importance the current FVs lack. A dual infection of FV-HIV in a human case has already been reported [75]. In addition, according to Murphy’s Law (“Whatever can go wrong *will* go wrong”), it is advisable to be prepared for a worst-case scenario. Thus, research and surveillance may aid in the early detection of recombination leading to novel viruses. 

The first evidence for homologous recombination in FVs *in vivo* came from the report by Liu *et al*. in which SFVs from chimpanzees were analyzed by single-genome amplification [37]. It was found that the original simian FV from chimpanzee (SFVcpz), which apes were infected with, recombined with SFVs of lower primates that the chimpanzees hunt for prey [37]. Furthermore, it was shown very recently by Galvin *et al*. [76] that SFVmcy-2 (better known as the laboratory strain *Macaca mulatta* SFV (SFVmac-2)) represents a recombinant form of SFVmcy-1 (SFVmac-1) and an African green monkey SFV (SFVagm (SFV-3))-like virus in the receptor-binding domain of *env* [76]. Furthermore, a cell culture model of FV recombination by template switching has been developed that indicated an almost 100% probability of the FV RT to switch to a closely-related template, provided both templates were expressed in the producer cells at equivalent levels [54]. However, so far, no clear picture has emerged from the considerable co-infection by different SFVs among NHPs in the wild [37,48] or in captivity [77], because in some monkeys, a recombinant virus appeared, and in others, the apparently peaceful coexistence of two strains took place [30,37,78]. In the event of persistently infected chimpanzees by lower primate FVs, the situation resembles somehow the reported zoonotic infections of humans by NHP FVs, except that there is no evidence for human-to-human transmission of FVs [79]. Given their overall common strategy for genome amplification [2,80], it is very unlikely, however, not completely formally excluded yet, that different FVs *in vivo* make use of different target cells (in particular, when heterologous hosts become infected). 

## 4. Retroviral Genome Diversity

**Figure 3:**
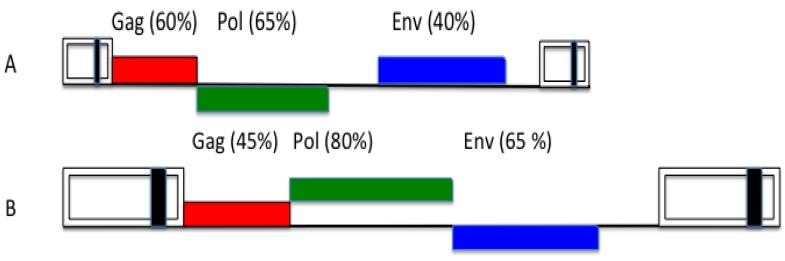
Overall nucleotide genome conservation of primate lentiviral (**A**) and primate foamy viral (**B**) genomes. The values are approximate and show a reciprocal feature of lentiviral and foamy viral *gag* and *env* genes. For clarity, accessory reading frames have been omitted (adapted from [81]).

The overall orthoretroviral genome diversity indicates that *env* genes are more variable than *gag*. The variability of lentiviral *envs* is well-known (Figure 3). FVs also appear to be an exception to this rule, because they show the opposite feature [81], since their *env* sequences are much more highly conserved than the respective *gag* genes (Figure 3). One hypothetical argument is that, probably, all FVs make use of the same cellular receptor superfamily [82,83] and must conserve recognizing epitopes. However, this argument is valid for HIV, as well. Another point concerns the FV replication strategy, in particular, the Env topology with leader peptide (LP), surface (SU) and transmembrane (TM) proteins that may exert a more rigid Env configuration and, consequently, sequence conservation of the FV tripartite than of the normal orthoretroviral bipartite protein [84]. However, this cannot be the whole story: e.g., why do we find conserved motifs in all FV LPs that are probably responsible for directly contacting cognate FV capsids [85,86], while the latter appear to show such a high variability?

A different aspect deals with the amino acid distribution in Gag of FVs. More or less all the basic residues in foamy viral capsid proteins are represented by arginines [87]. Lysine-poor viral proteins are very rare in virology [87]. The FV codon usage indicates that several of the arginine-specifying bases in contemporary *gag* genes were probably lysine-specifying in previous *gag* genes [87]. The analysis of PFV mutants, in which some arginine codons were mutated “back” to lysine codons, excluded a role of anti-retroviral restriction factors in lysine exclusion (see below). However, they showed a strong interferon (IFN) sensitivity as a possible explanation for lysine-poor Gag in FVs [87]. Thus, host IFN selection appears to be a potential long-term driving force shaping current FV Gags (see below). 

Since a functional explanation has not been defined yet, the distribution of variable and conserved motifs in FV genomes requires a closer look. Furthermore, it may be that some characteristic features of PFV and other Gags of primate FV origin, such as the nuclear pathway [88,89] or the intra-cellular retrotransposition [90,91], may turn out to be particular to PFV and close relatives, uncommon in other FVs and not actually representative of the FV-specific replication pathway [80,92].

## 5. Innate Host Defense and Viral Counter-Defense

Innate host defense and viral counter-defense can determine the outcome of many viral infections, resulting in the survival of hosts and viral genomes (positive selection). Central to the innate host defense is the interferon (IFN) system. Due to the profound effect of IFN on viral replication, there has been mutual evolutionary modulation by viruses on the host’s IFN system and by the host’s IFN system on viral genomes [93,94,95]. Thus, a constant arms race between viruses and hosts emerged [93,94], and retroviruses were no exception to the rule [93,94].

Early studies have indicated the sensitivity of FV replication to IFNs [96,97]. However, in these studies, FVs were regarded as very weak, if at all, inducers of an IFN response [96]. The vulnerability, in particular, to type II IFN, was later investigated from a very different angle, when it was shown that the addition of γ-antibodies greatly enhanced the chances to isolate FVs in cell culture from peripheral blood lymphocytes (PBLs) *ex vivo* [98] based upon that only 10–20 cells in 10^6^ PBLs appear to contain FV DNA on average [48,66,99]. In summary, these studies indicated the extreme vulnerability of FV replication to IFN, but left the question of IFN induction by FVs completely open. This was addressed in a more recent study by Rua *et al*. [48], who showed that as with other retroviruses [100,101], the toll-like receptor 7 (TLR7) is the main molecule in plasmacytoid dendritic cells (pDCs), the cells in the body that preferentially secret type I IFN [93], to sense FVs and to induce a strong IFN response [102]. TLR7 recognizes single-stranded RNA. Thus, although reverse transcription has commenced in the virus-producing cell far enough to make the virion DNA “infectious” [80,87,103,104], there is sufficient virion RNA present to trigger the IFN response. This strong host IFN response might indicate that natural infections by FVs were, in ancient times, not as harmless as they are apparently now. This scenario can be possibly mimicked by infection of specific IFN knockout mice. On the other hand, the vigorous IFN response may be a reason for the inert infection by FVs. 

The induction of IFNs may have resulted in various adaptations that allowed the virus to replicate even in their presence. The mode of action of IFNs to establish the antiretroviral state includes the hyper-induction of cellular proteins (restriction factors) by which the battle of host *vs*. virus begins. The restriction factors acting on retroviruses have been described in a number of excellent reviews [95,105,106,107]. Therefore, their mechanisms of action against orthoretroviruses will be mentioned here only when it is relevant to FV replication.

However, with FVs, it appears that the host-virus battle is supposedly over and may have occurred in evolutionary history. If FV infections were to be completely non-pathogenic, there would be no need for the host to adapt to these viruses nor for the virus to counteract the host response. In this situation the only “interest” of the virus would be to multiply its genome and propagate without affecting the host. This may be an additional reason for the initial difficulty in assigning Bet an APOBEC3-counteracting function [61] and for the finding of a *bet-*minus FV in humans [108]. However, we will leave this question open for the time being and will briefly look at the already described antiretroviral restriction factors:

(A) TRIM5α

The oligomeric tripartite motif (TRIM) proteins of primates include a large family of antiviral effectors that are made of conserved building blocks, namely an N-terminal RING finger domain, one or two B-box motifs, a coiled-coil motif (these three are collectively called the RBCC-domain) and the C-terminal B30.2/SPRY domain, which confers retrovirus capsid-binding [109,110].

TRIM5α proteins inhibit a very early stage of orthoretroviral replication before reverse transcription occurs by interfering with capsid breakdown and uncoating of the viral RNA, which is a requisite for reverse transcription to take place. To do this, they physically bind to capsids and might induce their proteasomal degradation [111,112,113,114,115]. However, the last point is dubious, since inhibition of the proteasome does not inhibit the anti-viral activity of TRIM5α [116,117]. Although regions and motifs in both proteins have been mapped [116,118], molecular details of how exactly TRIM5α can inhibit such diverse retroviruses are largely unknown. Very recently the crystallographic structure of an FV N-terminal Gag fragment has been resolved [119]. This analysis revealed not only clues about how FV Gag may interact with autologous Env, but also, how TRIM5α might bind to these unique retroviral capsids [119].

TRIM5α proteins act in a species-specific manner [116]. For instance, the rhesus macaque (mac) TRIM5α is active against HIV-1, and *vice versa*, human TRIM5α prevents an infection by SIVmac [116,118]. Since the effect of TRIM5α on viral replication is profound (up to two orders of magnitude are regularly observed in experimental settings [116,120]), the presence of TRIM5α has been discussed as a strong barrier against viral trans-species transmissions [116,118,121]. However, all TRIM5α proteins are inactive against the autologous virus, e.g., HIV-1 will replicate regardless of the presence or absence of human TRIM5α, because the retroviral capsid genes evolved to be insensitive to “their” TRIM5α proteins, arguing for a very long virus-host adaption.

FVs are also vulnerable to these proteins [120,122]. As with orthoretroviruses, the restriction is dictated by the B30.2 domain and exhibits some species-specificity [120], which suggests a more or less development by chance rather than having specific evolutionary implications. Moreover, concerning their protein and nucleic acid composition, FVs capsids are very much different from their orthoretroviral cousins [2,3]. Thus, the mechanism of TRIM5α action must be very much determined from a common structural motif, provided it is similar in both retroviral subfamilies. Since details on this mechanism have not been investigated for any of the spumaretroviruses, one cannot tell which evolutionary signature in contemporary foamy viral *gag* genes was imprinted by TRIM5α selection, except that such a signature must exist.

(B) APOBEC3

If the retroviral genomes were to be reverse transcribed (note that there is a distinctive difference between orthoretroviruses and spumaretroviruses with respect to the time-point of reverse transcription in the viral replication cycle, which, in the case of the latter, appears to take place to a very large extent in virus-producing cells [2,3]), intermediate RNA-DNA hybrids will be generated (Figure 1). The single-stranded DNA in these hybrids is the target of apolipoprotein B mRNA-editing enzyme catalytic polypeptide-like 3 (APOBEC3) proteins, which deaminate cytosines to uridines [106,123,124]. Furthermore, there is a deamination-independent APOBEC3 effect on the reverse transcription of lentiviruses [106,125,126,127]. It is worth mentioning here that there is a marked difference in how lentiviruses and spumaretroviruses react to prevent APOBEC3 packaging. Both virus genera generate a viral protein to antagonize APOBEC3. While in most lentiviruses, the Vif protein prevents APOBEC3 (mainly APOBEC3G and 3F) protein packaging by delivering them to the cellular proteasome for degradation [106,128], the foamy viral accessory Bet protein prevents encapsidation by blocking APOBEC3 dimerization and targeting them to form insoluble complexes incapable of incorporation into nascent FV capsids [129,130]. FV Bet proteins are not initiating APOBEC3 protein destruction [57,58,59].

Domains of physical interaction on the side of effector molecules and targets have been mapped [57,130]. Recent results from the feline FV system indicate that ORF-2 encodes the APOBEC3-interacting sequences [130]. On the other hand, the *tas*-exon of *bet* does not contribute directly in distracting APOBEC3; however, it may participate in the high *bet* expression level. 

The ORF-2 sequences evolutionarily shaped the genomic APOBEC3 locus and *vice versa* [57]. ORF-2/bet sequences belong to the more variable genomic regions of FVs and impose a high value of non-synonymous *vs*. synonymous mutations. This implies that, similar to the situation in the lentivirus genus [123,131], there is some species specificity in ORF-2/Bet to antagonize APOBEC3 proteins [57,129,130].

For lentiviruses, it has been proposed that the virus (HIV-1) benefits from a partially active APOBEC3 by exploiting this particular cellular mechanism of innate immunity to enhance viral diversity [132,133,134]. The strict conservation of their genomes illustrates that this is not an option for FVs. Moreover, the development of a viral protein to counteract APOBEC instead of evolving the *gag* genes, as a way not to package APOBECs, shows that either the latter is not so easily possible or that homologous-insensitive TRIM5α (see above) constitutes the older antiviral host activity. However, sequence evidence argues against the latter possibility [135]. Clearly, the clarification of this response needs further experimental approaches, in particular, the species specificity of FVs to antagonize APOBEC3 proteins requires further analysis. 

(C) Tetherin

From the viewpoint of virus replication, tetherin (CD317) is the last of the IFN-inducible factors that restrict FV replication, because it acts on the cell-free virion [136,137]. Tetherin is a type II trans-membrane protein with an N-terminal trans-membrane domain, followed by an extra-cellular domain that adopts a coiled-coil structure and a C-terminal glycosyl-phosphatidylinositol (GPI) anchor [107,138]. Due to its membrane-sticking features, tetherin inhibits the generation of free enveloped virions (not only form orthoretroviruses) [139,140]. 

Tetherin forms homodimers [107,138,141,142]. Dimerization is a requisite to act on lentiviruses; thereby, the Vpu or the Nef proteins have evolved to counteract and degrade tetherin [107,138,141,142]. In HIV-2, where a *vpu* ORF does not exist, the Env protein fulfils this function [107,138,141,142]. However, dimerization appears to be not essential for FV restriction, whereas the membrane insertion on both sides is [143]. FVs have no particular ORF to counteract tetherin. This feature might result in the low viremia and cell-associated nature of FV infections. Similar to FIV, which also does not contain a tetherin-antagonizing protein [144], this obviously does not prevent infections by FVs. 

(D) SamHD1

Weather the Sterile motif domain- and HD domain-containing protein 1 (SamHD1), which restricts HIV replication in myeloid cells by alteration of the nucleotide pool required for reverse transcription and which is counteracted by HIV-2 (and related viruses), Vpx protein [145] is also able to restrict FV replication and was investigated with a negative result recently [146]. Since SanHD1 acts on the nucleotide pool required for reverse transcription, it appeared *a priori* unlikely that it is able to restrict FVs, if added to target cells. Due to the feature of late reverse transcription, the FV genome already consists of DNA (Figure 1). 

## 6. On the Origin of PFV and Further Human FV Infections

**Figure 4:**
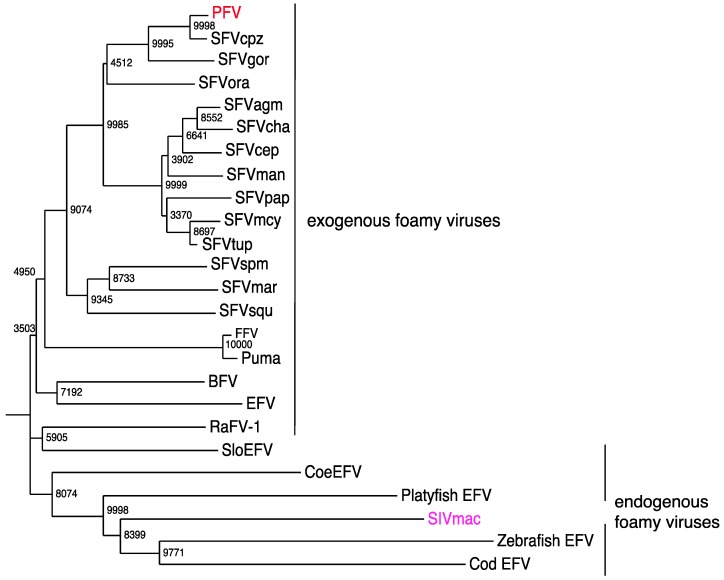
Phylogeny of current endogenous and exogenous FV *pol* amino acid sequences (from the active center-specifying integrase sequences and comprising 142 codons). A neighbor-joining tree was calculated by using the Maximum Composite Likelihood method with a bootstrap test of 10,000 replicates. BFV, bovine FV (NP_044929.1); EFV equine FV (NP_054716.1); PFV, prototypic FV (in red) (Y07725.1); SFVcpz, SFV from chimpanzee (CCP47057); SFVgor, SFV from gorilla (AY195688.1); SFVora, SFV from orangutan (CAD67562); SFVagm, African green monkey (*Cercopithecus aethiops*) FV (YP_001956722.2); SFVcha, SFV from *Chlorocebus aethiops* (CAM34599); SFVcep, SFV from *Cercopithecus pygerythrus* (AAV92627)*;* SFVman, SFV from mandrill (ADO65890.1); SFVpap, SFV from *Papio* (CAM34655); SFVmcy (previously SFVmac) from *Macaca mulatta*, SFV from *Macaca cyclopis* (CAA41394.1); SFVtup, SFV from *Tupaia* [155] (AGN49359); SFVspm, SFV from spider monkey (ABV59399.1); SFVmar, SFV from marmosets (ADE06000.1); SFVsqu, SFV from squirrel monkeys (ADE05995.1); FFV, feline FV (NP_056914); Puma, FV from *Puma concolor* (AGC11913); RaFV-1, FV from the bat *Rhinolophus affinis* (AFK85015); SloEFV, endogenous FV from sloths (Katzourakis *et al*., 2009); CoeEFV, endogenous FV in the *coelacanth* (*Latimeria*) genome (JX006241.1); platyfish EFV, endogenous FV from platyfish (M. Schartl, personal communication); zebrafish EFV, zebrafish (M. Schartl, personal communication); and Cod EFV, codfish (M. Schartl, personal communication) genomes; integrase encoding sequences of the macaque simian immunodeficiency virus (SIVmac in magenta) (AAC57420.1) served as the outlier; the endogenous FV sequence from aye-aye (PSFVaye) [156] was not incorporated, because integrase sequences are not available.

Early reports presented serological evidence of a human FV [147,148]. However, these studies could not be confirmed upon further investigations [149,150]. Furthermore, when an FV from chimpanzees was molecularly cloned and sequenced, it was suggested that the PFV isolate, which was obtained in 1970 from a Kenyan patient [151], might actually represent an ape virus [152] (Figure 4). 

**Figure 5:**
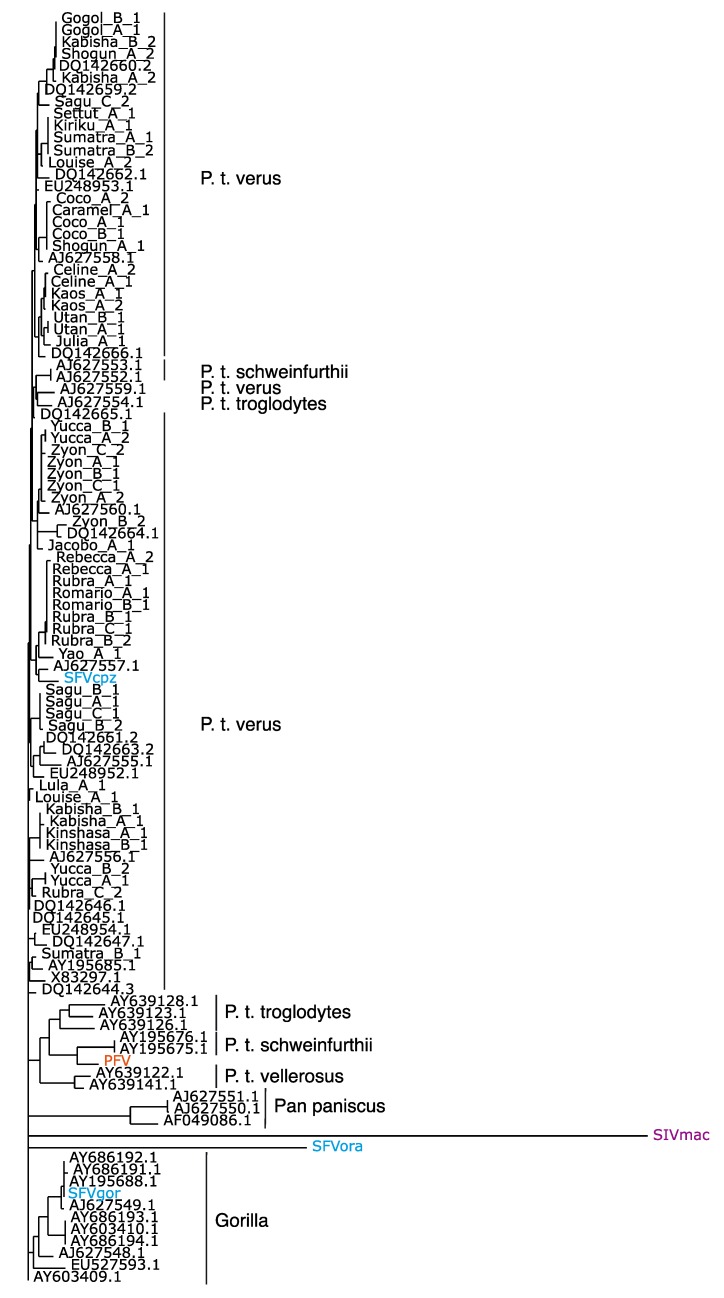
A 425-nucleotide fragment from the conserved region of the integrase domain of *pol* was used to establish the phylogenetic tree demonstrating the phylogenetic relations of SFVcpz, PFV and SFVgor. The position of the proviral PFV (red) sequence and other proviral plasmids (blue) are indicated. SIVmac (magenta) served as an outlier. *Pan paniscus* is Bonobo.

However, a more detailed evaluation, including DNA sequences, which became only available recently [48,50], reveals that the SFVs from chimpanzees present a wider sequence spectrum than the SFVs from gorillas (Figure 5). This finding represents only a very indirect support for the phylogeographic origin of FV sequences, because the actual geographic origin of the gorilla whose SFV sequence was reported first [48,50] is dubious. All other SFVgor sequences [48] were derived from animals stemming from a more limited area compared to the FVs derived from chimpanzees. These data are best compatible with the assumption of a strain-specific transmission pattern according to mother-to-child transmission and social groups [30]. In addition, they reflect behavioral differences between chimpanzees and gorillas, e.g., a more aggressive behavior of the former. Surely, more sequencing data from great ape FVs should reveal a better evolutionary relationship of FVs. However, PFV to SFV being almost identical in *P*.*t*. *schweinfurthii* points to these chimpanzee subspecies as a potential transmitter of this virus. 

Aside from PFV, there are many examples of human infections by NHP FVs among personnel in primate centers, zoos or other facilities housing NHPs or due to NHP biological materials [79]. Furthermore, African bushmeat hunters appear to be at high risk of SFV infections [48]. Another risk to acquire an FV infection exists for humans living close to Asian temple sites or other places, where humans often encounter free roaming monkeys [153]. In most cases, when humans acquired an FV infection, it was due to wounds in recipients and mostly from donor bites or deep scratches [48,79]. The infected humans remained unaware of their infection until—sometimes after decades [48,79]—researchers investigated their blood and were able to isolate virus in addition to finding serologic and nucleic acid evidence of infection [3,48]. However, retroviral infections sometimes go for long periods of time unnoticed and, in addition, pathological consequences may be rather subtle [154]; therefore, a careful follow up of the human cases is advisable. However, up to now, not a single case of human-to-human transmission has been reported. It appears as if mainly social behavior changes associated with human evolution were linked to a loss in FV susceptibility.

One point deserving further discussion is that FV transmitted from an NHP to a human did not yet evolve into a human FV, in sharp contrast to the development of HIV from simian immunodeficiency virus (SIV) transmitted to humans [175]. Even after decades of infection, the FV remained stable and maintained the genetic signature of the original infectious agent, *i*.*e*., the viral mutation rate in the new human host was not different from that in the natural ape or monkey host [48,79]. This is one of the clearest arguments for the non-pathogenic nature of FV infections. On the other hand, lessons from PTLV teach that the lack of human adaptation does not automatically preclude viral pathogenesis [70].

## 7. Exogenous FVs in the Animal Kingdom

The overall distribution of FV sequences indicates that these viruses are extremely widespread in the animal kingdom (Figure 4). FVs have not only been frequently found in NHPs of Old and New World origins, with a prevalence depending on the individual age and mounting up to 100% in grown up animals in the wild [30,37,77]. FVs are also present in prosimians, felines, bovines and equines [3,155,157,158]. Isolated reports on FVs from sheep [159], bison [160] and sea lions [161] suggest their presence in Bovidae and sea mammals. A notable exception is rodents, for which a specific FV has not been reported yet. Recently, Blasse *et al*. reported even the age-dependent infection of chimpanzees by more than one FV [30]. However, as to the extent of cross-neutralization of the superinfecting virus by immune response against the originally infecting FV, this has not been analyzed yet.

The various bat species are a major reservoir of viral infections, which may be eventually transmitted to humans as zoonoses; rhabdo-, corona-, filo-, orthomyxo-, paramyxo- and herpes-viruses are only some examples [162,163,164,165]. It came, therefore, as no major surprise that the detection of exogenous FV sequences upon screening of fecal samples from Asian bat species was reported recently by Wu *et al*. [166]. The finding of FVs in a such a diverse and old order illustrates the very old age of these viruses and asks for a more systematic screening and molecular characterization of FVs isolated from animals (not necessarily only mammals; see below) close to evolutionary branch points or otherwise interesting to science.

## 8. Endogenous FVs

If retroviruses get access to gametocytes, the result can be an endogenous form of the virus, which than mutates “neutrally” and is passaged in the germline along with the host cell genome and no longer transmitted as an exogenous virus [167,168]. In the vast majority of cases, “lethal” mutations accumulate over time in replication-required viral reading frames [167]. Approximately 8% of the human genome is made up by endogenous retroviruses, and a further 30%–40% of sequences are the result of reverse transcription [167]. 

Based on some sequence homology, the endogenous retrovirus-like (ERV) family L has been proposed to represent distantly related human and animal endogenous FVs [169]. However, the stretch of homology is rather short and does not hold up for the rest of the viral genome [170]. Thus, it was almost two decades before researchers again looked for endogenous FVs. They came up with some interesting findings from several pretty much different species. In any case, they demonstrated the very old age of FVs.

Firstly, Katzourakis *et al*. [171] reported their presence in the two- and three-toed sloths from South America (SloEFV) (Figure 4). Both species were separated approximately 21 million years ago (MYA). Since these endogenous elements were absent in other *Xenarthrans* species (e.g., ant bears and armadillos), which separated from the sloths 55 MYA, the authors conclude the establishment of SloEFV during this period (55 MYA–21 MYA) and the previous presence of exogenous FVs [171].

Secondly, Han and Worobey published [156] an endogenous FV sequence (PSFVaye), which was derived from the Madagascar aye-aye lemur (*Daubentonia madagascarensis*), a primitive prosimian. This suggests that exogenous FVs were present before the split of *Strepsirrhini* (mainly galagos, loris and lemurs) and *Haplorhini* (mainly the simians of the Old and New World) around 85 MYA, because the aye-aye is phylogenetically basal to the current lemurs [156]. 

Thirdly, it was reported again by Han and Worobey [172] that, surprisingly, the *Coelacanth* (*Latimeria*) genome harbors endogenous FV-like elements (CoeEFV) (Figure 4). *Latimeria* is a “living fossil”, believed to be a side branch of those now extinct lobe-finned fish, from which all current land vertebrates developed. If FVs were indeed to be present in these species, the FV-host coevolution represents an age of over 400 MYA, by far oldest known virus-host relationship. For instance, they would be approximately 30-times older than the oldest endogenous lentivirus we know [173]. 

Fourthly, Schartl *et al*. recently reported the discovery of fish endogenous FVs from the platyfish (*Xiphophorus*) and the cod species [174]. Furthermore, FV-like sequences were previously described in the zebra fish genome [175]. These discoveries do not necessarily extend the age of FVs; rather, the finding in another vertebrate order, in addition to mammals, supports the notion of the remarkable evolutionary success of this retroviral subfamily. Not all fish species harbor endogenous FVs [174], and the finding of almost intact copies of some FV genes [174], that is, without the accumulation of “lethal” mutations, might indicate the recent evolutionary invasion from exogenous FV.

## 9. Conclusions

1. Exogenous FVs might be present in species outside the class of placental Mammalia. Therefore, it will be scientifically extremely interesting to see FV isolates/sequences from Marsupialia, monotremes, birds, reptiles and amphibians. Even their presence as exogenous viruses in some fish species is not excluded, and the very much cell-associated nature of the agents might have yet prevented their detection.

2. The very long coevolution with animals may be the real reason for the benign nature of FV infections. For reasons of scientific consistency, it might be theoretically distracting and, for reasons of vector application, probably, medically beneficial that a genuine human FV does apparently not exist.
